# Optimization of levetiracetam dosing regimen in critically ill patients with augmented renal clearance: a Monte Carlo simulation study

**DOI:** 10.1186/s40560-022-00611-w

**Published:** 2022-04-21

**Authors:** Idoia Bilbao-Meseguer, Helena Barrasa, Alicia Rodríguez-Gascón, Eduardo Asín-Prieto, Javier Maynar, José Ángel Sánchez-Izquierdo, María Ángeles Solinís, Arantxazu Isla

**Affiliations:** 1 Pharmacokinetic, Nanotechnology and Gene Therapy Group (PharmaNanoGene), Faculty of Pharmacy, Centro de Investigación Lascaray Ikergunea, University of the Basque Country UPV/EHU, Paseo de la Universidad 7, 01006 Vitoria-Gasteiz, Spain; 2 Bioaraba, Microbiology, Infectious Disease, Antimicrobial Agents, and Gene Therapy, Vitoria-Gasteiz, Spain; 3 Department of Pharmacy, Cruces University Hospital, Plaza de Cruces 12, Barakaldo, 48903 Bizkaia, Spain; 4 Bioaraba, Intensive Care Unit, Vitoria-Gasteiz, Spain; 5 Osakidetza Basque Health Service, Araba University Hospital, Intensive Care Unit, c/ Olaguibel no. 29, Vitoria-Gasteiz, Spain; 6 Inserm U1070: Pharmacologie des anti-infectieux, Pôle Biologie Santé-Bâtiment B36, Université de Poitiers, 1 rue Georges Bonnet, 86022 Poitiers, France; 7 Intensive Care Unit, Doce de Octubre Hospital, Avda de Córdoba, s/n, 28041 Madrid, Spain; 8 Present Address: PharmaMar, Avda. De los Reyes, 1, Pol. Ind. La Mina-Norte, Colmenar Viejo, 28770 Madrid, Spain

**Keywords:** Levetiracetam, Dosing, Pharmacokinetics, Pharmacodynamics, Monte Carlo simulation, Augmented renal clearance, Critically ill patient, Neurocritical care

## Abstract

**Background:**

Levetiracetam pharmacokinetics is extensively altered in critically ill patients with augmented renal clearance (ARC). Consequently, the dosage regimens commonly used in clinical practice may not be sufficient to achieve target plasma concentrations. The aim of this study is to propose alternative dosage regimens able to achieve target concentrations in this population. Furthermore, the feasibility of the proposed dosing regimens will be discussed from a clinical point of view.

**Methods:**

Different dosage regimens for levetiracetam were evaluated in critically ill patients with ARC. Monte Carlo simulations were conducted with extended or continuous infusions and/or high drug doses using a previously developed population pharmacokinetic model. To assess the clinical feasibility of the proposed dosages, we carried out a literature search to evaluate the information on toxicity and efficacy of continuous administration or high doses, as well as the post-dilution stability of levetiracetam.

**Results:**

According to the simulations, target concentrations in patients with CrCl of 160 or 200 mL/min can be achieved with the 3000 mg daily dose by prolonging the infusion time of levetiracetam. For patients with CrCl of 240 mL/min, it would be necessary to administer doses higher than the maximum recommended. Available evidence suggests that levetiracetam administration in continuous infusion or at higher doses than those approved seems to be safe. It would be desirable to re-examinate the current recommendations about drug stability and to achieve a consensus in this issue.

**Conclusions:**

Conventional dosage regimens of levetiracetam (500–1500 mg twice daily in a short infusion) do not allow obtaining drug plasma concentrations among the defined target in critically ill patients with ARC. Therefore, new dosing guidelines with specific recommendations for patients in this subpopulation are needed. This study proposes new dosages for levetiracetam, including extended (4 or 6 h) infusions, continuous infusions or the administration of doses higher than the recommended in the summary of product characteristics (> 3000 mg). These new dosage recommendations take into account biopharmaceutical and pharmacokinetic aspects and meet feasibility criteria, which allow them to be transferred to the clinical environment with safety and efficacy. Nevertheless, further clinical studies are needed to confirm these results.

## Background

Since levetiracetam was introduced in Europe, it has become a very frequently used antiseizure medication in the intensive care units (ICUs). It is used both in the treatment of focal and generalized onset seizures, and in the second line treatment of status epilepticus. Moreover, despite the lack of a robust recommendation, levetiracetam has been increasingly used in the ICUs in many clinical scenarios (after craniotomy, subarachnoid hemorrhage (SAH) or traumatic brain injury (TBI)) due to its relative ease of use, efficacy, and low side effects profile [1]. There is no clear differentiation between prophylactic and therapeutic doses. Thus, in general, it is recommended to start with 500 mg twice daily and increase the dose until the therapeutic effect is achieved up to a maximum of 1500 mg twice daily. This could justify that the most frequently used dose in prophylaxis is 500 mg/12 h [2].

The altered pathophysiology in critically ill patients can have a major impact on the pharmacokinetic parameters of drugs [3–5]. One of the phenomena in this population that is gaining relevance is augmented renal clearance (ARC). ARC is defined as a creatinine clearance (CrCl) > 130 mL/min/1.73 m^2^. It is present in 20–65% of critically ill patients with younger age, polytrauma and lower severity illness being identified as risk factors [6]. Furthermore, it seems to be more common in certain situations, such as TBI and SAH, clinical conditions that usually justify the use of anticonvulsants either prophylactically or therapeutically [7–10]. The presence of ARC in critically ill patients has been consistently associated with subtherapeutic antimicrobial plasma concentrations and it may have a negative impact on the attainment of therapeutic levels of many drugs [11–15]. Although its influence has been studied mainly in the context of antimicrobial therapy, ARC has the potential to influence the pharmacokinetic profile of any drug that is renally cleared and known to have a direct correlation between their renal clearance (CL) and CrCl, such as levetiracetam.

The reference range for levetiracetam trough concentrations is 12–46 mg/L at steady state, as recommended by the International League Against Epilepsy (ILAE) [16]. To date, several studies on levetiracetam in critically ill patients indicate that the dosages regimens commonly used are not sufficient to achieve plasma concentrations within this range, specifically in critically ill patients with ARC [17–20].

These results are in line with a recently published systematic review and meta-analysis (30 studies, *n* = 7609 patients), which assesses the use of levetiracetam compared with no antiseizure medication or with a different antiseizure medication for the prevention of first seizure across neurocritical patients [2]. They could not demonstrate significant reductions in seizure incidence and, neither support nor refute the use of levetiracetam prophylaxis in TBI, SAH, intracerebral hemorrhage or supratentorial neurosurgery. However, their data suggested that levetiracetam might be superior to other seizure medications following supratentorial neurosurgery. They hypothesized that the use of low-dosage levetiracetam, with 500 mg twice daily being the most common dosage used across the studies, might not generate therapeutic levels. These results suggested the need to establish new dosage guidelines that allow reaching the therapeutic objective in this population.

Therefore, the aim of this study is to put forward alternative dosage regimens, using stochastic simulations, able to achieve target concentrations in critically ill patients with ARC receiving levetiracetam. Furthermore, the feasibility of the proposed dosing regimens will be discussed from a clinical point of view considering the potential toxicity and efficacy of the doses and mode of administration evaluated, as well as the stability of the pharmaceutical preparation.

## Methods

### Optimized dosage regimen proposals for critically ill patients with ARC in treatment with levetiracetam

New dosage regimens for levetiracetam were simulated in critically ill patients with ARC (CrCL of 160, 200 and 240 mL/min). Dosing proposals include the use of continuous infusion, extended infusion times (4 or 6 h) and/or the administration of increasing doses (from 3000 mg up to 6000 mg daily). Stochastic dosing simulations were performed by a population pharmacokinetic model (PPK) recently published by our group [17]. This PPK model was developed from a multicentric open-label prospective study conducted in 27 critically ill patients treated with levetiracetam and with a CrCl > 50 mL/min (range 54–239 mL/min) measured in urine. The model is described in Table 1.

**Table 1:** Population pharmacokinetic model used in the simulations

Parameter	Model estimate (RSE (%))
CL (L/h) = θnr + (CrCl/120)^θr^	–
θnr	3.5 (9)
θr	2.5 (17)
V1 (L)	20.7 (18)
Q (L/h)	31.9 (22)
V2 (L)	33.5 (13)
IIV_CL (%)	32.7 (21)
IIV_V1 (%)	56.1 (29)
RE_proportional (%)	22.3 (15)

*CL* clearance, *CrCl* creatinine clearance, *V1* central volume of distribution, *Q* intercompartmental clearance, *V2* peripheral volume of distribution, *IIV* inter-individual variability, *RE* residual error, *RSE* relative standard errors

Monte Carlo simulations were performed in NONMEN^®^ (v.7.4) to generate the concentration–time profiles in 1000 virtual subjects. The percentiles of steady-state trough concentrations by the simulated dosing regimens were subsequently determined in R (v.4.0.2). The probabilities of achieving target trough concentrations were estimated for the reference range of 12–46 mg/L.

### Evaluation of dosage regimens feasibility

To assess the clinical feasibility of proposed dosages of levetiracetam, we carried out an evaluation of the following aspects: (1) evidence of toxicity or efficacy of extended or continuous administration mode, (2) evidence of toxicity or efficacy of high doses and (3) stability issues.

To gather information on these aspects, two tertiary databases, UpToDate^®^ [21] and Micromedex^®^ [22] were consulted. In addition, to evaluate the extended or continuous infusion mode, a bibliographic search was carried out in MEDLINE, from inception until October 2021. The following terms were used: (“levetiracetam” OR “keppra”) AND (“extended” OR “continuous”) AND “infusion”. For stability evaluation three electronic drug compatibility references (King Guide^®^ to Parenteral Admixtures^®^ [23], Trissel's 2 Clinical Pharmaceutics Database^®^ [24] and Stabilis^®^ database [25]) and manufacturers' online labeling were also consulted [26–28]. Finally, other references considered to be relevant were identified in a non-systematic literature search.

## Results

### Optimized dosage regimen proposals for critically ill patients with ARC in treatment with levetiracetam

Table 2 summarizes the probabilities of target attainment (PTA), that is, the percentage of virtual patients that maintained trough drug concentrations at steady state above 12 mg/L and below 46 mg/L.

**Table 2:** Probability of target attainment based on Monte Carlo simulations

CrCl (mL/min)	Total daily dose (mg)	Dose (mg)	Dosing interval (h)	Perfusion duration (h)	Probability of Cmin (%)	
					> 12 mg/L	> 46 mg/mL
160	3000	1500	12	0.5	51	0
				4	62	< 0.5
				6	70	< 0.5
		1000	8	0.5	65	0
				4	**81**	< 0.5
				6	**88**	1
		3000	24	24	**98**	1
	4500	1500	8	0.5	**89**	5
200	3000	1000	8	6	69	< 0.5
		3000	24	24	**89**	< 0.5
	4500	1500	8	4	**84**	1
				6	**92**	2
	6000	2000	8	0.5	**84**	5
240	3000	3000	24	24	68	< 0.5
	4500	1500	8	4	61	< 0.5
				6	74	< 0.5
		4500	24	24	**96**	1
	6000	2000	8	4	**80**	2
				6	**89**	3
		6000	24	24	**99**	7

*Cmin* minimum levetiracetam concentration, *CrCl* creatinine clearance. In bold, PTA (probability of Cmin higher than 12 mg/L) > 80%

Based on our simulations, for patients with CrCl of 160 mL/min, it would be possible to achieve a PTA of at least 80% with 1000 mg infused over 4 h every 8 h or with 1500 mg over 30 min every 8 h. For patients with CrCl of 200 mL/min, it would be necessary to administer 3000 mg in continuous infusion, 1500 mg over 4 h every 8 h or 2000 mg over 30 min every 8 h. For patients with CrCl of 240 mL/min, it would be necessary to administer 4500 mg in continuous infusion or 2000 mg over 4 h every 8 h. With those dosing regimens, the probability of Cmin to exceed the value of 46 mg/L is < 5%.

### Evaluation of dosing regimens feasibility

#### Mode of administration: extended or continuous infusion

Currently, there is experience on the use of levetiracetam in continuous infusion, both intravenously and subcutaneously. Overall, although more studies would be necessary, levetiracetam given as a continuous infusion appears to be effective and well tolerated.

Our search identified two publications that include patients receiving intravenous levetiracetam in continuous infusion. Moodle et al. [29], made a retrospective study of 36 patients with diagnosis of status epilepticus and who had been treated with intravenous levetiracetam. Thirty patients received levetiracetam as bolus infusions and 6 as continuous infusion. Efficacy was higher if a bolus was administered compared with continuous infusions without initial loading bolus (*p* = 0.002). The aim of the study was not to investigate the differential efficacy of both methods of administration and plasma levels were not measured. Nevertheless, authors hypothesized that in the context of status epilepticus peak levels after rapid levetiracetam infusions might be responsible for higher effectiveness of bolus compared with continuous pump infusions. No severe adverse effects related to levetiracetam infusion were described and treatment was overall well tolerated. Burakgazi et al. [30] published a retrospective study with 33 patients who received intravenous levetiracetam (16 as bolus and 17 as continuous infusion) for treatment or prevention of seizures with the aim of discussing its safety and tolerability. They concluded that intravenous levetiracetam, regardless of the method of administration, was not associated with any adverse events in hospitalized patients.

There are also case reports assessing the administration of levetiracetam in subcutaneous continuous infusion in the context of palliative care. In this setting, levetiracetam subcutaneous infusion seems to be an effective option for seizure control with good adverse effect profile [31–34]. However, randomized controlled trials are needed to establish the efficacy and tolerability of subcutaneous levetiracetam administration.

Micromedex^®^ [22] includes the study of Burakgazi et al. [30] in its information, while UpToDate^®^ [21] does not make references to this method of administration in its monograph of levetiracetam.

#### The use of high doses

The information contained in the summary of product characteristics (SPC) establishes a maximum dose of 3000 mg per day [26, 27], based on phase III trials with fixed dose regimens. Even that the evaluation of a dose–effect relationship was not the primary objective of these trials, the results give an indication of a dose–effect relationship in this dose range [35–37].

However, for higher doses (up to 4000 mg) it has been considered that they did not increase efficacy but increased the rate of side effects [38, 39]. This is based on studies that compared differing levetiracetam fixed doses according to a group comparison. A more recent retrospective study [40], which included 61 patients treated with levetiracetam, analyzed individual response to a levetiracetam dose increment. It concluded that dose escalation improved treatment outcomes without additional safety hazards. The final daily doses ranged from 1000 to 6000 mg.

In tertiary databases [21, 22], the maximum dose recommended in the treatment of focal and generalized onset seizures or prophylactically it is also 3000 mg per day.

#### Stability of levetiracetam infusion solutions

According to the European SPC of Keppra^®^ [26], intravenous levetiracetam is physically compatible and chemically stable for at least 24 h at room temperature. In the case of the SPC authorized by FDA [27], the information was the same until 2016, when it was modified. Currently it states that the diluted solution should not be stored for more than 4 h at controlled room temperature. However, there are other FDA-approved levetiracetam medications that maintain 24-h stability and there are also pre-diluted alternatives [28].

The information regarding stability of levetiracetam solutions found in the consulted electronic databases is scarce and differs between them. While in King Guide to Parenteral Admixtures^®^ [23], a 24 h at room temperature stability is granted, Trissel's 2 Clinical Pharmaceutics Database^®^ [24] only gives a stability of 4 h at room temperature based on the SPC of Keppra^®^ authorized by FDA. Stabilis^®^ database [25] does not provide information on stability at room temperature.

## Discussion

This study is, to the best of our knowledge, the first to propose alternative dosing regimens for levetiracetam in critically ill patients with ARC. Dosing simulations suggest the need to administer up to 6000 mg of levetiracetam daily to reach the target plasma level. Our results indicate that it is necessary to optimize the dosage regimen in terms of increasing the dose and/or infusion time to reach the target plasma concentrations in this group of patients. Considering this evidence, it is worth wondering whether we are using levetiracetam adequately in critically ill patients, especially in those with ARC. This should be an issue to be taken into account in daily clinical practice, because ARC has been identified in 20–65% of ICU patients and in up to 85% of neurocritical patients [6–10].

Currently, the reference range for levetiracetam trough concentrations has been stablished by the ILAE in 12–46 mg/L [16]. However, studies carried out in critically ill patients have shown that these plasma concentrations are not achieved with the authorized adult dosing regimen. To date, four PPK studies of levetiracetam have been identified in neurocritical care patients. Spencer et al. [19] included 12 adult patients who received levetiracetam. They estimated a higher levetiracetam CL and a shorter half-life compared with previously published results in healthy volunteers. Just one patient, with renal impairment (CrCl 42 mL/min), achieved a steady-state trough concentration greater than 6 mg/L. Sime et al. [18] developed a population pharmacokinetics model in 30 critically ill patients with severe TBI or SAH without renal dysfunction. For every 40 mL/min/1.73 m^2^ increase in urinary CrCl, levetiracetam CL increased by 50% and the median trough concentrations were reduced by 50%. They performed dosing simulations with dosages ranging from 1000 mg every 12 h to 2000 mg every 8 h and concluded that for urinary CrCl greater that 120 mL/min/1.73 m^2^, none of the simulated regimens had a probability of 80% or above of achieving trough concentrations higher than 12 mg/L. Similarly, Ong et al. [20] developed a PPK model in 20 neurosurgical patients. They also performed Monte Carlo simulations showing a low probability of reaching trough concentrations > 6 mg/L with the 500 mg twice daily dosing regimen. Finally, our group also reported a population pharmacokinetic model in 27 critically ill patients [17], not restricted to neurocritical patients. CrCl demonstrated a significant influence on the levetiracetam CL. Dosing simulations showed that the administration of at least 500 mg every 8 h or 1000 mg every 12 h would be needed in patients with normal renal function and that higher doses or shorter dosing interval would be needed in patients with ARC.

According to these PPK models, the dosage regimen of 500 mg every 12 h is insufficient to achieve a PTA of at least 80% in ICU patients with a normal renal function. However, this is a widely used dosage in clinical practice, especially in the prophylactic context, where between 34 and 100% of patients received this dosage [17–20]. Furthermore, the maximum dosage approved for levetiracetam, 3000 mg daily in short infusion, also resulted in subtherapeutic levels in patients with ARC. Our results confirm that the target plasma levels would only be reached in ARC patients with the administration of at least 3000 mg in 4-h infusion (in patients with CrCl of 160 mL/min) or in continuous infusion (in patients with CrCl of 200 mL/min). Although extended and continuous infusions are not included in the SPC of levetiracetam, they may be an alternative that avoids the use of doses higher than 3000 mg. However, in patients with CrCl of 240 mL/min, it is not possible to reach the target plasma levels with the maximum authorized dose regardless of the mode of administration, and higher doses are compulsory.

For an adequate management of these patients, however, the ARC should be considered as a dynamic and temporary situation and, consequently, patients' renal function should be assessed daily to adjust dosing regimens if necessary [6, 16]. Equations that estimate glomerular filtration rate have been shown to be inappropriate in critically ill patients [41], and specifically in patients with ARC as they tend to underestimate the value of CrCl in this population [6]. For this reason, creatinine clearance measured in urine should be the routine technique for calculating CrCl in ICU patients, and this value should be used to adjust the dosing regimens of drugs affected, such as levetiracetam.

Several factors are needed to be considered before considering applying in the clinical practice these results obtained by means of pharmacokinetic simulations, that is, the feasibility of the proposed dosage strategies must be pondered from different approaches. In the case of levetiracetam, there is sufficient experience to consider safe its administration in prolonged infusions [29–34]. However, it is important to take into account that extended infusions do not allow reaching therapeutic levels from the beginning of the treatment; therefore, in patients who were not undergoing previous treatment with the drug, it is necessary to consider a loading dose. Considering levetiracetam Vd is not affected by patient`s CrCl, the required loading dose would be the same as in patients without ARC (1000–1500 mg). On the other hand, it should be noted that the administration in extended or continuous infusion makes sense in situations in which we want to maintain stable drug levels in the blood for prolonged time. Therefore, these strategies would not be suitable for example in the acute treatment of status epilepticus, where high single dose bolus is usually recommended (1 to 3 g at a rate of 2 to 5 mg/kg/min or 40 to 60 mg/kg as a single dose infused over 5–15 min in combination with a parenteral benzodiazepine, and with a maximum dose of 4.5 g) [21]. Finally, one potential drawback to prolonged or continuous infusion is the need for a venous access site in patients with limited lumens available.

The safety of administering doses higher than those authorized in the SPC must be considered. Our dosing simulations suggest the need to administer up to 6000 mg of levetiracetam daily to reach the target plasma level. To date, available evidence shows a good safety profile with the use of high doses of levetiracetam [40]. Nevertheless, the objective of our simulations is to reach levels within the therapeutic range in a group of patients in which, due to their characteristics, the clearance of the drug is increased. For this reason, the use of high doses in this context can be considered safe, although it is necessary to closely monitor patients and, if possible, perform therapeutic monitoring of the drug.

Finally, when administering a drug in extended or continuous infusion, the information on drug stability is critical. Indeed, short post-dilution stability can prevent the drug from being administered in this way. However, different stabilities have been set for levetiracetam by different regulatory agencies, which can condition the proposal of new dosage regimens. On the one hand, EMA [20] accepted that levetiracetam is stable for at least 24 h at room temperature; on the other hand, FDA [21] limited it to 4 h. This discrepancy might suppose the use of extended and prolonged perfusions impossible under FDA criteria, whereas feasible in Europe. Therefore, it would be desirable to re-examinate the current recommendations about drug stability and to achieve an international consensus regarding this issue.

Although this research reached its aims, it has certain limitation: first of all, there is a limited number of PPK studies of levetiracetam including ARC condition and all the results are obtained from simulations based on a previously published study carried out in a relatively small population, which included patients with CrCL > 50 mL/min, but only 37% had ARC. Second, the objective of our simulations was to evaluate the adequacy of currently levetiracetam dosage regimens to achieve plasma levels within the range established by the ILAE. However, there is a lack of consensus about which the target concentrations for levetiracetam treatment are, and no specific target has been defined in prophylactic use. Although, the dosage regimens used in prophylactic context are usually the same as those listed for seizure treatment and the majority of clinical trials in which the efficacy of levetiracetam in prophylaxis has been evaluated use same guidelines, the relationship between levetiracetam plasma levels and its efficacy or toxicity needs to be further characterized in both situations. That is, even if there are studies that analyze the influence of the ARC in the achievement of plasma levels within the currently accepted range, there is no data linking this situation with higher incidence of seizures. Therefore, further investigations overcoming these limitations are needed to confirm these results in the clinical setting.

## Conclusions

This study states that conventional dosage regimens do not allow obtaining drug plasma concentrations among the therapeutic range of levetiracetam in critically ill patients with ARC, and highlights the need to implement new dosing guidelines that include specific recommendations for patients in this subpopulation. The recommended regimens must take into account biopharmaceutical and pharmacokinetic aspects that condition the probability of treatment success, such as the controversial stability of the drug in solution or the duration of perfusion. We proposed new dosage recommendations, to be implemented in critically ill patients with ARC, which meet feasibility criteria that allow them to be transferred to the clinical environment with safety and efficacy. According to simulation results, sometimes extended or continuous infusions would be needed, and in other situations, it would be necessary to administer doses higher than those authorized. Nevertheless, further clinical studies are needed to confirm these results.

## Abbreviations

ARC: Augmented renal clearance
CL: Clearance
CrCl: Creatinine clearance
ICU: Intensive care unit
ILAE: International League Against Epilepsy
PPK: Population pharmacokinetic model
PTA: Probability of target attainment
SAH: Subarachnoid hemorrhage
SPC: Summary of product characteristics
TBI: Traumatic brain injury

## Declarations

### Availability of data and materials

The data sets used and/or analyzed during the current study are available from the corresponding author on reasonable request. The population PK model used for simulations has been published at: Bilbao-Meseguer et al. [17].
